# Molecular residual disease and efficacy of adjuvant chemotherapy in patients with colorectal cancer

**DOI:** 10.1038/s41591-022-02115-4

**Published:** 2023-01-16

**Authors:** Daisuke Kotani, Eiji Oki, Yoshiaki Nakamura, Hiroki Yukami, Saori Mishima, Hideaki Bando, Hiromichi Shirasu, Kentaro Yamazaki, Jun Watanabe, Masahito Kotaka, Keiji Hirata, Naoya Akazawa, Kozo Kataoka, Shruti Sharma, Vasily N. Aushev, Alexey Aleshin, Toshihiro Misumi, Hiroya Taniguchi, Ichiro Takemasa, Takeshi Kato, Masaki Mori, Takayuki Yoshino

**Affiliations:** 1 Department of Gastroenterology and Gastrointestinal Oncology, National Cancer Center Hospital East, Kashiwa, Japan; 2 Department of Surgery and Science, Graduate School of Medical Sciences, Kyushu University, Fukuoka, Japan; 3 Translational Research Support Office, National Cancer Center Hospital East, Kashiwa, Japan; 4 The Second Department of Internal Medicine Center, Osaka Medical and Pharmaceutical University, Takatsuki, Japan; 5 Division of Gastrointestinal Oncology, Shizuoka Cancer Center, Sunto-gun, Japan; 6 Department of Surgery, Gastroenterological Center, Yokohama City University Medical Center, Yokohama, Japan; 7 Gastrointestinal Cancer Center, Sano Hospital, Kobe, Japan; 8 Department of Surgery 1, School of Medicine, University of Occupational and Environmental Health, Kitakyushu, Japan; 9 Department of Gastroenterological Surgery, Sendai City Medical Center Sendai Open Hospital, Sendai, Japan; 10 Division of Lower GI Surgery, Department of Gastroenterological Surgery, Hyogo Medical University, Hyogo, Japan; 11 Oncology, Natera, Inc., Austin, TX USA; 12 Department of Data Science, National Cancer Center Hospital East, Kashiwa, Japan; 13 Department of Clinical Oncology, Aichi Cancer Center, Nagoya, Japan; 14 Department of Surgery, Surgical Oncology and Science, Sapporo Medical University, Sapporo, Japan; 15 Department of Surgery, National Hospital Organization Osaka National Hospital, Osaka, Japan; 16 Tokai University School of Medicine, Isehara, Japan

**Keywords:** Colorectal cancer, Prognostic markers

## Abstract

Despite standard-of-care treatment, more than 30% of patients with resectable colorectal cancer (CRC) relapse. Circulating tumor DNA (ctDNA) analysis may enable postsurgical risk stratification and adjuvant chemotherapy (ACT) treatment decision-making. We report results from GALAXY, which is an observational arm of the ongoing CIRCULATE-Japan study (UMIN000039205) that analyzed presurgical and postsurgical ctDNA in patients with stage II–IV resectable CRC (*n* = 1,039). In this cohort, with a median follow-up of 16.74 months (range 0.49–24.83 months), postsurgical ctDNA positivity (at 4 weeks after surgery) was associated with higher recurrence risk (hazard ratio (HR) 10.0, *P* < 0.0001) and was the most significant prognostic factor associated with recurrence risk in patients with stage II or III CRC (HR 10.82, *P* < 0.001). Furthermore, postsurgical ctDNA positivity identified patients with stage II or III CRC who derived benefit from ACT (HR 6.59, *P* < 0.0001). The results of our study, a large and comprehensive prospective analysis of ctDNA in resectable CRC, support the use of ctDNA testing to identify patients who are at increased risk of recurrence and are likely to benefit from ACT.

## Abstract (web summary)

In the observational GALAXY arm of the CIRCULATE-Japan study, among 1,049 patients with stage II–IV colorectal cancer, those with presence of circulating tumor DNA (ctDNA) after tumor resection have a high recurrence risk and are likely to benefit from adjuvant chemotherapy.

## Main

Surgical resection is the standard-of-care for patients with stage II or III colorectal cancer (CRC), with subsequent adjuvant chemotherapy (ACT) performed based on clinicopathological risk factors^1,2^. For clinical stage IV or relapsed CRC cases with oligometastasis, perioperative chemotherapy with metastasectomy is considered. However, over 30% of patients with stage II or III CRC and 60–70% of patients after oligometastatic resection experience recurrence^3,4^. The American Society of Clinical Oncology (ASCO) panel states that the current definition of ‘high-risk’ stage II cancer is inadequate as many patients with high-risk features do not experience recurrence, whereas some patients with average-risk features do. In addition, the ASCO panel acknowledges that none of the listed high-risk features are predictive of benefit from ACT^5,6^. However, the current National Comprehensive Cancer Network guidelines have remained in place since 2004 owing to a lack of better prognostic factors to guide treatment decisions. Although ACT has been shown to improve survival in patients with stage III colon adenocarcinoma and is recommended for all patients with stage III colon adenocarcinoma who are eligible to receive chemotherapy, researchers have reported large variability in outcomes and questioned the absolute benefit of ACT in this population. In an analysis of 12,834 patients with stage III colon cancer enrolled in the IDEA trial, the 5-year disease-free survival (DFS) rate varied greatly among subgroups, ranging from 89% for the lowest-risk stage III (T1N1a) group to 31% for the highest-risk cohort (T4N2b). The absolute DFS gain of ACT for the lowest-risk stage III group and the highest-risk group was 8% and 20%, respectively^7^. Therefore, it is reasonable to assume that a substantial portion of patients with low-risk stage III cancer can safely forgo ACT or be considered for treatment de-escalation (3 months versus 6 months). Circulating tumor DNA (ctDNA) can be a powerful tool to help guide treatment decisions in this cohort, especially for patients with borderline performance status for whom treatment toxicity is a main concern.

ctDNA is a minimally invasive biomarker that can aid in the measurement of disease status across several settings, including postcurative surgery or treatment for detection of molecular residual disease (MRD), during surveillance, and throughout the course of therapy for treatment response monitoring. The value of tumor-informed ctDNA analysis has been evaluated in several retrospective and prospective studies in patients with early-stage CRC. Among patients with early-stage CRC, ctDNA positivity after curative-intent surgery has been associated with higher rates of disease recurrence^8^.

To prospectively validate and build upon the previously published evidence, we sought to demonstrate that postsurgical ctDNA positivity (MRD time point) is predictive of disease recurrence in early-stage CRC. The GALAXY study, which is a part of the CIRCULATE-Japan project, is a large prospective, observational study that monitors ctDNA status for patients with clinical stage II to IV or recurrent CRC following curative-intent surgery^9^. In this analysis, we report on postsurgical ctDNA positivity and its associations with patient outcomes and its implications for ACT selection, and the association between ctDNA dynamics and prognosis.

## Results

### Patient characteristics

**Fig. 1:**
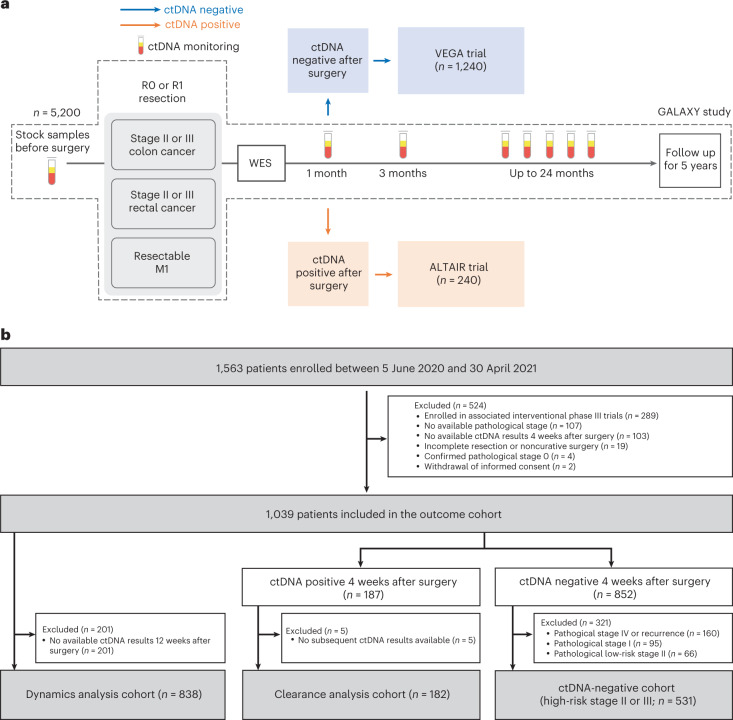
Study design and population. **a**, Overview of CIRCULATE-Japan study, illustrating the observational GALAXY protocol with sample collection schema. **b**, CONSORT (Consolidated Standards of Reporting Trials) diagram illustrating patient inclusion and exclusion criteria for sub-analyses.

Of the 1,039 patients included in the ctDNA analysis, 18.0% (187 out of 1,039) were ctDNA positive 4 weeks after surgery, and 82.0% (852 out of 1,039) were ctDNA negative. To evaluate ctDNA dynamics from 4 weeks to 12 weeks, patients who experienced recurrence within 12 weeks (*n* = 45) or who did not have ctDNA status available 12 weeks after surgery (*n* = 157) were excluded, and the remaining 838 patients were analyzed. For clearance analysis, among patients who were ctDNA positive 4 weeks after surgery (*n* = 187), those with no available ctDNA status 12 weeks after surgery were excluded (*n* = 5), and the remaining 182 were analyzed (Fig. 1b). Patient characteristics are summarized along with ctDNA status at the 4-week postsurgical time point in Table 1. There were significant differences in sex, primary location, pathological stage, *RAS* and *BRAF* status, and microsatellite instability (MSI) status between the ctDNA-positive and ctDNA-negative groups (Table 1).

**Table 1:** Patient characteristics and ctDNA status at 4 weeks after surgery

Patient characteristics	Category	ctDNA negative (*n* = 852), *n* (%)	ctDNA positive (*n* = 187), *n* (%)	*P* value^a^
Age (years)	Median (range)	69 (25–93)	67 (39–88)	
	≤ 70	478 (56.1)	115 (61.5)	0.18
	>70	374 (43.9)	72 (38.5)	
Sex	Male	433 (50.8)	117 (62.6)	0.003
	Female	419 (49.2)	70 (37.4)	
ECOG performance status	0	744 (87.3)	165 (88.2)	0.33
	1	108 (12.7)	22 (11.8)	
Primary location	Right-sided colon	349 (41.1)	53 (28.6)	0.001
	Left-sided colon	452 (53.2)	113 (61.1)	
	Rectum	48 (5.7)	19 (10.3)	
Pathological T stage	T1–T2	117 (15.9)	7 (5.0)	<0.001
	T3–T4	619 (84.1)	133 (95.0)	
Pathological N stage	N0	402 (54.7)	34 (24.3)	<0.001
	N1–N2	333 (45.3)	106 (75.7)	
Pathological stage	Stage I	95 (11.1)	2 (1.1)	<0.001
	Stage II	291 (34.1)	24 (12.8)	
	Stage III	306 (36.0)	91 (48.7)	
	Stage IV or recurrence	160 (18.8)	70 (37.4)	
*RAS* and *BRAF* status	*RAS* and *BRAF* wild-type	422 (49.5)	94 (50.3)	0.02
	*RAS* mutant	352 (41.3)	87 (46.5)	
	*BRAF* mutant	78 (9.2)	6 (3.2)	
MSI status	MSI-high	95 (11.2)	5 (2.7)	<0.001
	MSS	757 (88.8)	182 (97.3)	

ECOG, Eastern Cooperative Oncology Group; MSS, microsatellite stable.

^a^*P* values were calculated using a two-sided chi-squared test comparing the distribution of the factors between the two columns (ctDNA positive versus ctDNA negative) with no correction for multiplicity.

### Personalized ctDNA assay based on tumor tissue-based WES

**Extended Data Fig. 1:**
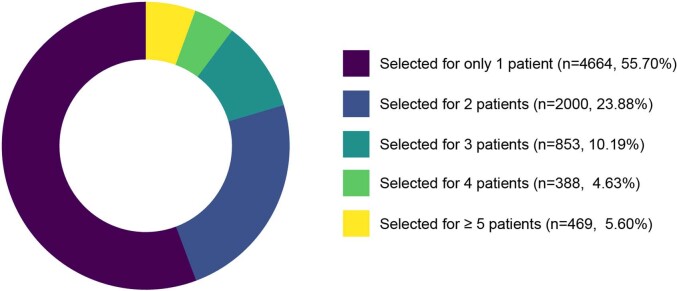
Genes Selected for ctDNA assay. A total of 8,374 genes were selected for 1,039 patients.

**Extended Data Fig. 2:**
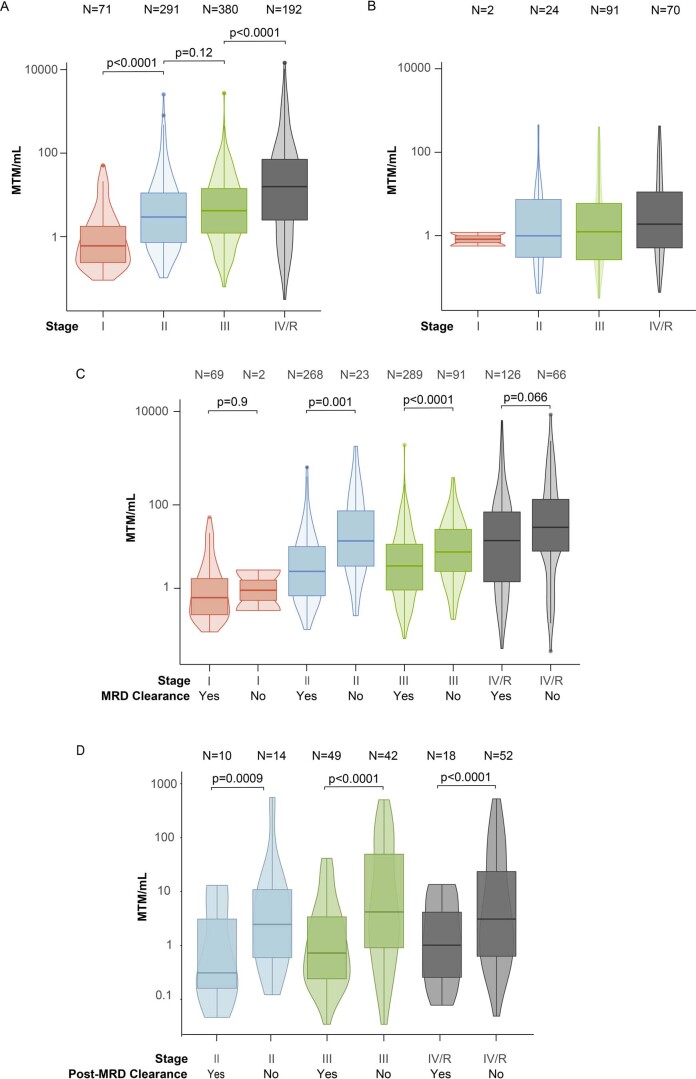
ctDNA is Elevated in MRD-Positive Patients. Average ctDNA concentration, in mean tumor molecules/mL of plasma (MTM/mL), across stages (stage I (red, stage II (blue), stage III (green), and stage IV (grey) at the following time points was assessed at: **A**. Prior to surgery, **B**. 4 weeks post-surgery (MRD timepoint), **C**. Patients who cleared their ctDNA at the MRD time point were observed to have lower presurgical ctDNA levels. **D**. Patients were further stratified by ACT induced ctDNA clearance beyond the 4-weeks post-surgical MRD time point across stages. Patients who cleared ctDNA during ACT had lower ctDNA at the MRD time point. Box plots were generated using ggplot2 package v3.3.6 in R v4.2.1. Center line, median MTM/ml; bottom of box, 25% quantile; top of box, 75% quantile; data points represent outliers. P-values were calculated using the two-sided t-test.

Whole exome sequencing (WES) was performed, and results were analyzed to design tumor-informed, personalized ctDNA assay. A total of 8,374 genes were selected for 1,039 patients. The most frequently selected genes were *TP53* (25.6%) and *APC* (17.5%). More than 50% of genes were unique to each patient, suggesting large variability in the mutational landscape of CRC outside of known hotspot regions (Extended Data Fig. 1). Among patients with ctDNA positivity, median ctDNA concentrations before surgery varied according to pathological stage (stage I, median 0.60 mean tumor molecules (MTM) per ml; stage II, 2.94 MTM per ml; stage III, 4.16 MTM per ml; and stage IV or recurrent disease, 15.74 MTM per ml) (Extended Data Fig. 2a). No significant differences in postsurgical ctDNA concentrations were observed across stages (Extended Data Fig. 2b). Interestingly, patients who had a higher ctDNA concentration before surgery were more likely to be ctDNA positive 4 weeks after surgery, regardless of pathological stage (Extended Data Fig. 2c). Furthermore, patients treated with ACT who had higher ctDNA concentrations 4 weeks after surgery were less likely to have ctDNA clearance at any subsequent time point, suggesting that the extent of molecular disease burden may be a factor in determining ACT efficacy (Extended Data Fig. 2d).

### Association of 4-week postsurgical ctDNA status with DFS

**Fig. 2:**
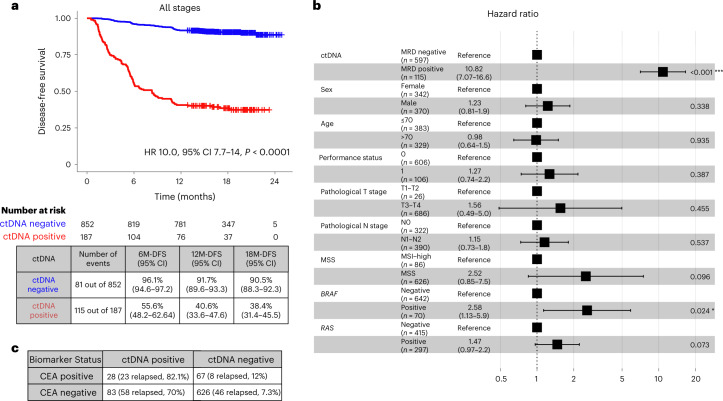
ctDNA-based MRD testing is predictive of survival outcomes in postsurgical patients with CRC. **a**, Kaplan–Meier estimates for DFS stratified by ctDNA-negative and ctDNA-positive status 4 weeks after surgery (pathological stages I–IV). HRs and 95% CIs were calculated using the Cox proportional hazard model. *P* values were calculated using the two-sided log-rank test. 6M-DFS, 6-month DFS; 12M-DFS, 12-month DFS; 18M-DFS, 18-month DFS. **b**, Forest plot depicting the multivariate analysis for recurrence in patients with pathological stage II–III CRC. Various prognostic factors and their association with DFS, as indicated by HR, were analyzed across the cohort using the two-sided Wald chi-squared test. The unadjusted HRs (squares) and 95% CIs (horizontal lines) are shown for each prognostic factor. Vertical dotted line, the null hypothesis. Number of events = 102; global *P* value (log-rank) = 2.4188 × 10^−27^; Akaike information criterion = 1,183.72; concordance index = 0.81. **c**, Correlation between ctDNA status and CEA status 12 weeks after surgery.

**Extended Data Fig. 3:**
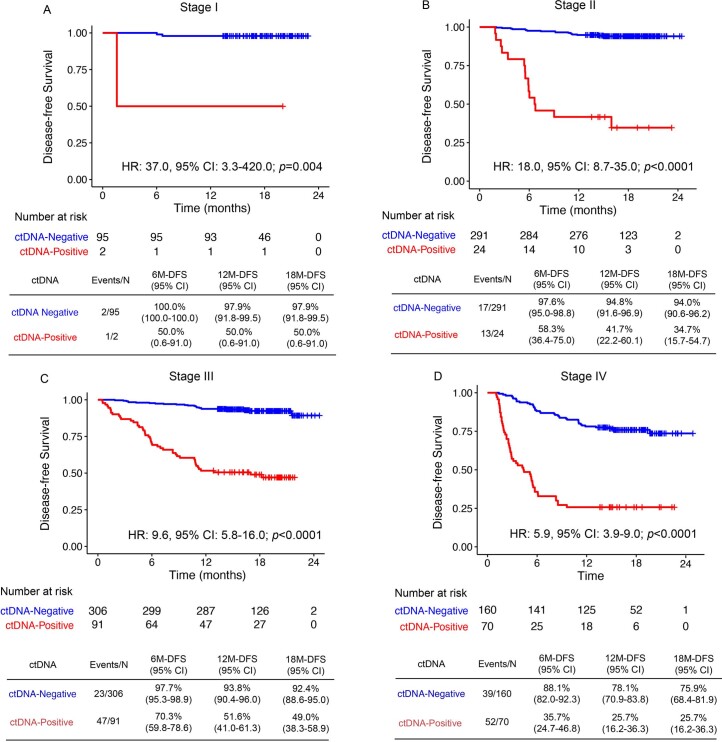
ctDNA-based MRD testing is Prognostic of Survival Outcomes in Post-Surgical CRC Patients. Kaplan-Meier estimates for disease-free survival stratified by ctDNA-negative (blue) and ctDNA-positive (red) status at 4-weeks post-surgery in patients by pathological stage: **A**. Stage I, **B**. Stage II, **C**. Stage III, and **D**. Stage IV CRC. Hazard ratio (HR) and 95% confidence interval (CI) were calculated by the Cox proportional hazard model. P-value was calculated using the two-sided log-rank test.

**Extended Data Fig. 4:**
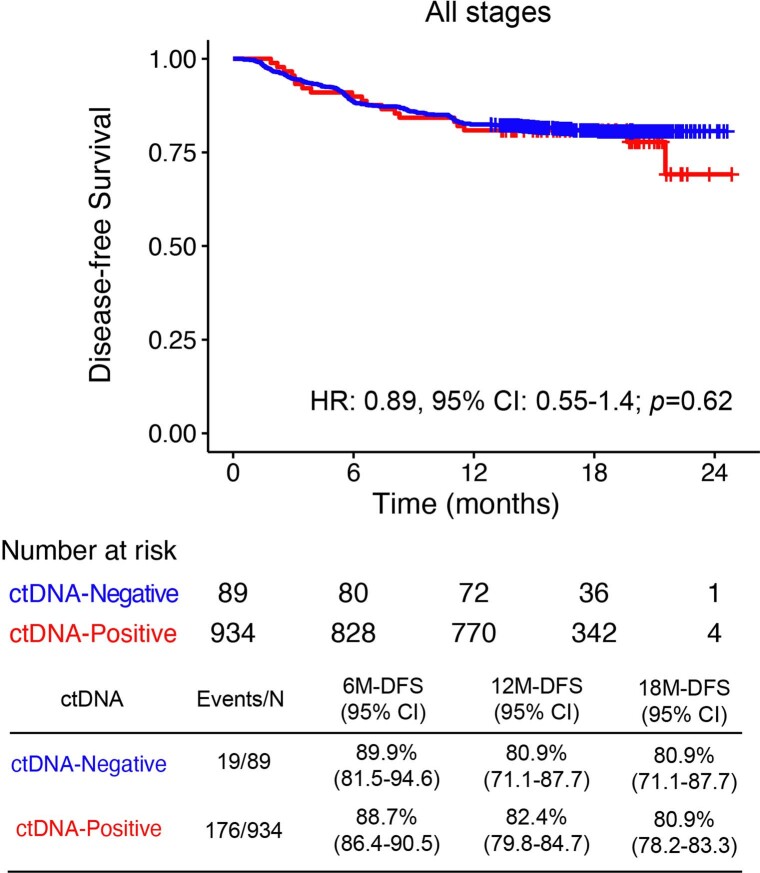
Presurgical ctDNA-based MRD testing is not Prognostic of Survival Outcomes in CRC Patients. Kaplan-Meier estimates for disease-free survival stratified by ctDNA-negative (blue) and ctDNA-positive (red) status before surgery. Hazard Ratio (HR) and 95% confidence interval (CI) were calculated by the Cox proportional hazard model. P-value was calculated using the two-sided log-rank test.

As of 8 June 2022, the median follow-up period was 16.74 months (range 0.49–24.83 months). Among 187 patients who were ctDNA positive 4 weeks after surgery, 61.4% (115 out of 187) experienced recurrence, whereas only 9.5% (81 out of 852) of patients who were ctDNA negative 4 weeks after surgery experienced recurrence (hazard ratio (HR) 10.0, 95% confidence interval (CI) 7.7–14.0, *P* < 0.0001), demonstrating an 18-month DFS of 38.4% (95% CI 31.4–45.5%) versus 90.5% (95% CI 88.3–92.3%), respectively (Fig. 2a). This trend was observed across all pathological stages: stage I (HR 37.0, 95% CI 3.3–420.0, *P* = 0.004) (Extended Data Fig. 3a); stage II (HR 18.0, 95% CI 8.7–35.0, *P* < 0.0001) (Extended Data Fig. 3b); stage III (HR 9.6, 95% CI 5.8–16.0, *P* < 0.001) (Extended Data Fig. 3c); and stage IV (HR 5.9, 95% CI 3.9–9.0, *P* < 0.0001) (Extended Data Fig. 3d). By contrast, no significant difference in recurrence risk by presurgical ctDNA status was observed (HR 0.89, 95% CI 0.55–1.4, *P* = 0.62) across all stages (Extended Data Fig. 4). In multivariate analysis for DFS in patients with pathological stage II–III disease, ctDNA positivity 4 weeks after surgery was the most significant prognostic factor associated with increased risk for recurrence (HR 10.82, 95% CI 7.07–16.6, *P* < 0.001) (Fig. 2b). Of note, all clinicopathological risk factors traditionally used for staging and prognostication were nonsignificant.

Furthermore, as per the protocol, carcinoembryonic antigen (CEA) data were available 12 weeks after surgery for a set of patients. We compared postsurgical (12-week) CEA status with postsurgical (12-week) ctDNA status and observed a concordance of ctDNA positivity and negativity in 81.3% (654 out of 804) of patients and a discordance in 18.7% (150 out of 804) of patients. Among the discordant cases, 70% (58 out of 83) of the ctDNA-positive (CEA-negative) patients relapsed, compared with 12% (8 out of 67) of the CEA-positive (ctDNA-negative) patients (Fig. 2c). These data suggest that ctDNA was more informative than CEA for relapse detection.

### Association of ACT with 4-week postsurgical ctDNA status and DFS

**Fig. 3:**
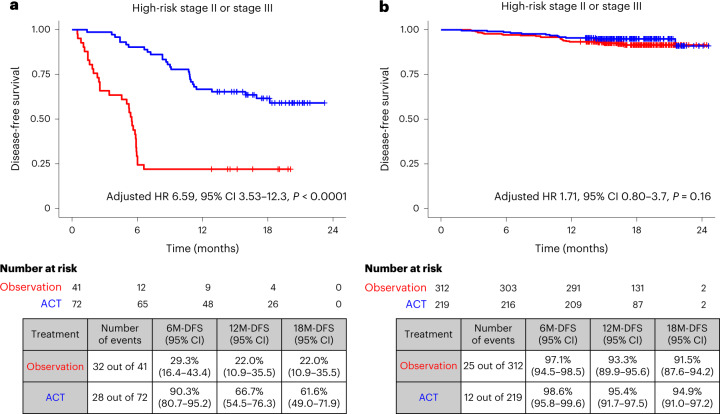
ctDNA-based MRD testing is predictive of response to ACT in postsurgical patients with CRC. **a**,**b**, Kaplan–Meier estimates for DFS stratified by observation and ACT in patients with high-risk pathological stage II or stage III disease and ctDNA positivity 4 weeks after surgery (**a**) (HR was adjusted by sex and performance status) and ctDNA negativity 4 weeks after surgery (**b**) (HR was adjusted by age, pathological stage, MSI and performance status). HRs and 95% CIs were calculated using the Cox proportional hazard model. *P* values were calculated using the two-sided log-rank test.

**Extended Data Fig. 5:**
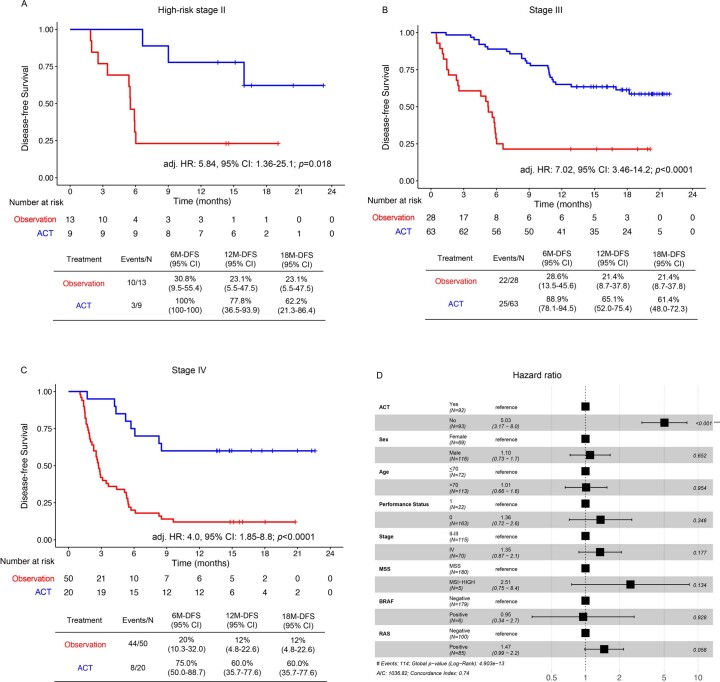
ctDNA-based MRD testing is Predictive of Response to Adjuvant Chemotherapy in Post-Surgical Patients with Colorectal Cancer. Kaplan-Meier estimates for disease-free survival stratified by observation (red) and adjuvant chemotherapy (blue) in patients with ctDNA positivity at 4-week after surgery, by stage: **A**. High-risk stage II patients; **B**. Stage III patients **C**. Stage IV patients. Hazard ratio (HR) was adjusted by sex, and performance status. Hazard ratio and 95% confidence interval (CI) were calculated by the Cox proportional hazard model. P-value was calculated using the two-sided log-rank test **D**. Forest plot depicting the multivariate analysis for recurrence in pathological-stage II-IV ctDNA-positive patients. Various prognostic factors and their association with disease-free survival, as indicated by hazard ratio were analyzed across the cohort using two-sided Wald chi-square test. The unadjusted hazard ratio (squares) and 95% CIs (horizontal lines) are shown for each prognostic factor. Vertical dotted line indicates the null hypothesis.

Given that ACT treatment is recommended for patients with high-risk stage II disease and all patients with stage III disease based on the standard of care, we examined the outcomes of ctDNA-positive and ctDNA-negative patients stratified by ACT status. To decrease the chance of disease and patient-related bias, we adjusted for confounding variables such as age, sex, MSI, pathological stage, and performance status in this analysis. We found that patients with high-risk stage II or stage III disease and ctDNA-positive status 4 weeks after surgery derived significant benefit from ACT (adjusted HR 6.59, 95% CI 3.53–12.3, *P* < 0.001) (Fig. 3a), and the trend was observed across all pathological stages: high-risk stage II (adjusted HR 5.84, 95% CI 1.36–25.1, *P* = 0.018); stage III (adjusted HR 7.02, 95% CI 3.46–14.2, *P* < 0.0001); and stage IV (adjusted HR 4.0, 95%CI 1.85–8.8, *P* < 0.0001) (Extended Data Fig. 5a–c). Furthermore, in a multivariate analysis of ctDNA-positive patients with stage II–IV disease, lack of ACT was found to be the most significantly negative prognostic factor (adjusted HR 5.03, 95% CI 3.17–8.0, *P* < 0.001) (Extended Data Fig. 5d). Interestingly, three out of four ctDNA-positive patients with pathological stage I or low-risk stage II disease did not receive ACT and experienced recurrence.

**Extended Data Fig. 6:**
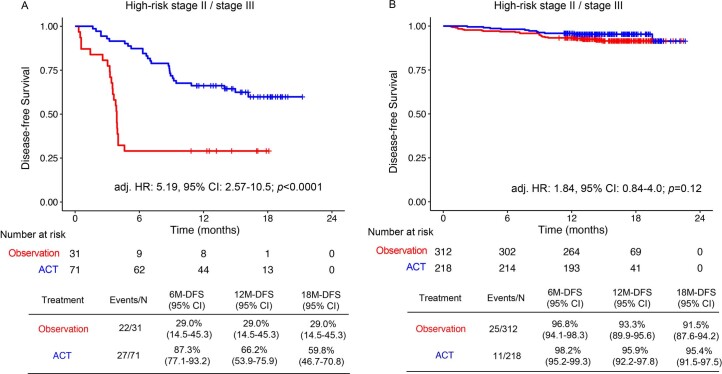
Landmark analysis at 8 weeks representing ctDNA-based MRD testing to be Predictive of Response to ACT in Post-Surgical CRC Patients. Kaplan-Meier estimates for disease-free survival stratified by observation (red) and adjuvant chemotherapy (blue) in pathological high-risk stage II/Stage III CRC patients with **A**. ctDNA positivity at 4-weeks after surgery; HR was adjusted by sex, and performance status. **B**. ctDNA negativity at 4-weeks after surgery; HR was adjusted by age, p-stage, MSI and performance status. A landmark analysis at 8 weeks was implemented to address the immortal time bias, whereby DFS was measured starting from day 60. Hazard ratio (HR) and 95% CI were calculated by the Cox proportional hazard model. P-value was calculated using the two-sided log-rank test.

**Extended Data Fig. 7:**
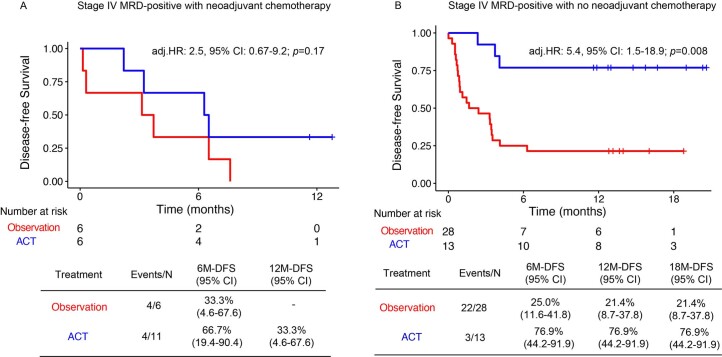
ctDNA-based MRD testing is Predictive of Response to ACT in Oligometastatic stage IV ctDNA-positive patients who did not receive neoadjuvant chemotherapy. Kaplan-Meier estimates for disease-free survival stratified by observation (red) and adjuvant chemotherapy (blue) in oligometastatic stage IV patients with **A**. ctDNA positive patients who received neoadjuvant therapy; HR was adjusted for gender. **B**. ctDNA-positive patients who did not receive neoadjuvant chemotherapy; HR was adjusted for gender and performance status. A landmark analysis at 8 weeks was implemented to address the immortal time bias, whereby DFS was measured starting from day 60. HR and 95% CI were calculated by the Cox proportional hazard model. P-value was calculated using the two-sided log-rank test.

Conversely, among 531 patients with high-risk pathological stage II or stage III disease and ctDNA-negative status 4 weeks after surgery, 41.2% (219 out of 531) of patients received ACT. Single-agent capecitabine (which is a fluoropyrimidine) and capecitabine in combination with oxaliplatin-based therapy were administered to 34 (15.5%) and 185 (84.5%) patients, respectively. In ctDNA-negative patients, after accounting for potentially confounding factors (age, sex, pathological stage, MSI and performance status), no statistically significant benefit from ACT was observed (adjusted HR 1.71, 95% CI 0.80–3.7, *P* = 0.167). Furthermore, an 18-month DFS of 94.9% (95% CI 91.0–97.2%) and 91.5% (95% CI 87.6–94.2%) was observed for the ACT group and the observation (no-ACT) group, respectively (Fig. 3b). Supplementary Tables 1 and 2 present the baseline characteristics of patients with high-risk stage II and stage III disease with ctDNA-positive and ctDNA-negative results 4 weeks after surgery, respectively, further stratified by ACT status. In addition, we implemented a landmark analysis 8 weeks after surgery to address the immortal time bias, whereby DFS was measured starting from day 60. A similar trend was observed, in which ctDNA-positive patients with high-risk stage II or stage III disease derived greater benefit from ACT (adjusted HR 5.19; 95% CI 2.57–10.5, *P* < 0.0001) (Extended Data Fig. 6a) than did ctDNA-negative patients (adjusted HR, 1.84, 95% CI 0.84–4.0, *P* = 0.129) (Extended Data Fig. 6b). Furthermore, we analyzed the effect of ACT in MRD-positive patients with oligometastatic stage IV CRC who did and did not receive neoadjuvant chemotherapy (NAC) and found that ACT was not associated with a significant improvement in DFS in patients who received NAC (adjusted HR 2.5, 95% CI 0.67–9.2, *P* = 0.17) (Extended Data Fig. 7a). Meanwhile, ACT resulted in a more pronounced improvement in DFS in patients who underwent upfront surgical resection (without NAC) (adjusted HR 5.4, 95% CI 1.5–18.9, *P* = 0.008) (Extended Data Fig. 7b).

### Association of postsurgical ctDNA dynamics from week 4 to week 12 with DFS

**Fig. 4:**
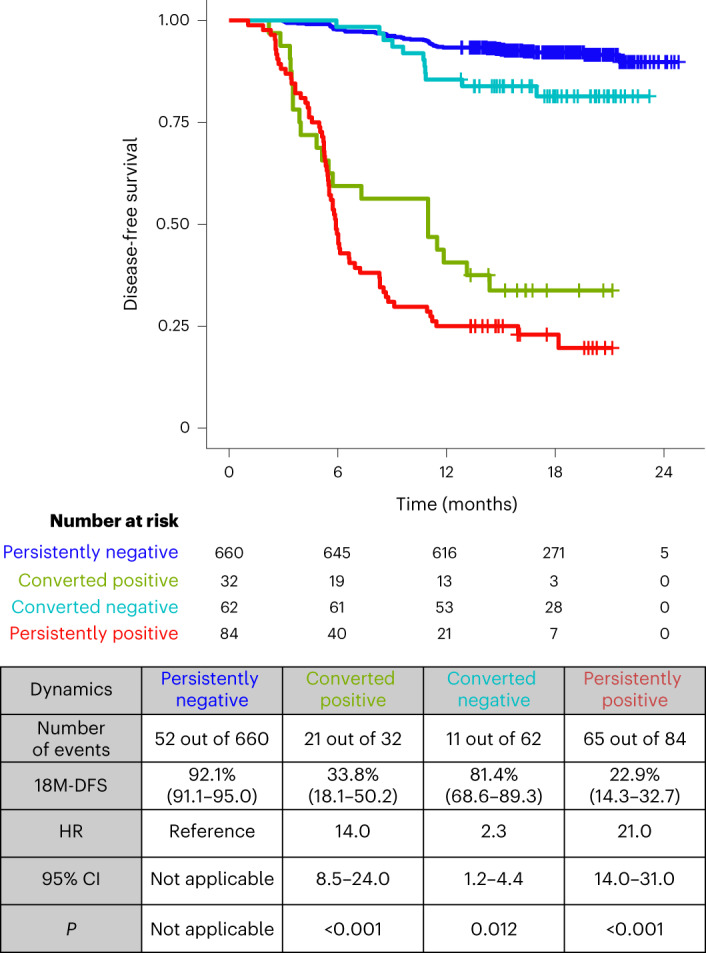
ctDNA-based treatment response monitoring is possible in postsurgical patients with CRC. Kaplan–Meier estimates for DFS according to ctDNA dynamics from 4 weeks to 12 weeks after surgery. Landmark analysis was performed 12 weeks after surgery. HRs and 95% CIs were calculated using the Cox proportional hazard model. *P* values were calculated using the two-sided log-rank test.

Among 838 patients with available ctDNA status both 4 weeks and 12 weeks after surgery, 42.2% (*n* = 354) received ACT. We implemented a landmark analysis to address the immortal time bias to account for the fact that patients must have lived at least 12 weeks to be included in this analysis. Of the total patients, 10% (*n* = 84 out of 838) stayed ctDNA positive and 78.8% (*n* = 660 out of 838) stayed ctDNA negative, whereas 3.8% (*n* = 32 out of 838) converted from negative to positive and 7.4% (*n* = 62 out of 838) converted from positive to negative (Fig. 4). Compared with the risk of recurrence for patients who were persistently negative, a significantly higher risk of recurrence was observed for patients who converted from ctDNA negative to positive (HR 14.0, 95% CI 8.5–24.0, *P* < 0.001), with an 18-month DFS of 33.8% (95% CI 18.1–50.2%), and for patients who remained persistently positive (HR 21.0, 95% CI 14.0–31.0, *P* < 0.001), with an 18-month DFS of 22.9% (95% CI 14.3–32.7%) (Fig. 4).

### Association of ACT with ctDNA clearance and DFS

**Fig. 5:**
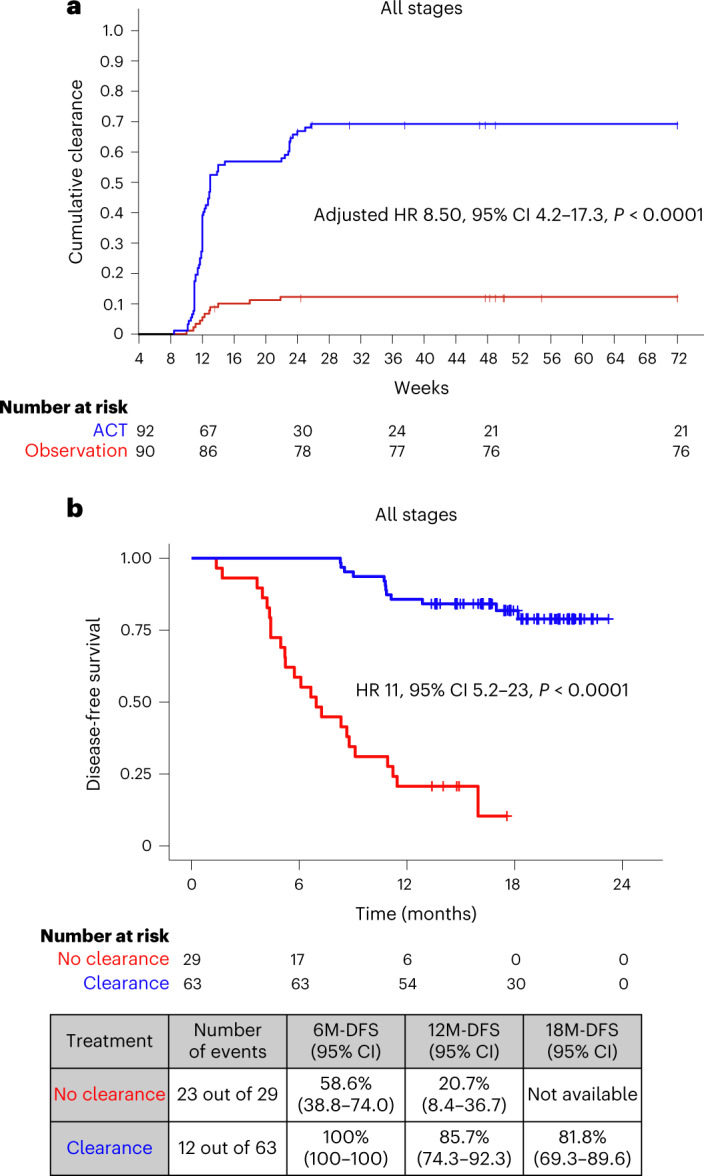
Cumulative incidence of ctDNA clearance in patients with pathological stage I–IV CRC. **a**, Kaplan–Meier estimates indicating cumulative clearance in patients stratified by ACT and observation. Differences in cumulative incidence were investigated using two-sided Gray’s test. **b**, Kaplan–Meier estimates for DFS for ctDNA-positive patients 4 weeks after surgery according to their ctDNA clearance status on ACT. HRs and 95% CIs were calculated using the Cox proportional hazard model. *P* values were calculated using the two-sided log-rank test. HR was adjusted by sex, performance status and pathological stage.

For ctDNA clearance analysis, we defined clearance as the time elapsed from the week-4 positive ctDNA result until the date of the first ctDNA-negative result, irrespective of subsequent ctDNA status. For this analysis, patients who remained MRD positive and did not miss a visit before relapse were considered to have never achieved clearance. Of the 187 patients with ctDNA positivity 4 weeks after surgery, 182 had ctDNA clearance data available. Of the 182 patients, 92 received ACT and 90 were kept in the observation arm (no-ACT). Patient characteristics among those treated with and those treated without ACT are shown in Supplementary Table 3. In this analysis, ACT was associated with a higher estimated cumulative incidence of ctDNA clearance in 68.48% (63 out of 92) of patients by week 24 after surgery compared with 12.2% (11 out of 90) of patients who did not receive ACT (adjusted HR 8.50, 95% CI 4.2–17.3, *P* < 0.0001; HR was adjusted for sex, age, stage, MSI and performance status) (Fig. 5a). This observed difference in cumulative clearance was statistically significant (*P* < 0.001) based on Gray’s test. Furthermore, on comparing the ctDNA clearance status of MRD-positive patients who were treated with ACT, we found that patients who did not achieve clearance exhibited an inferior DFS (adjusted HR 11, 95% CI 5.2–23.0, *P* < 0.0001) (Fig. 5b).

## Discussion

MRD, which is defined as ctDNA positivity after curative surgery or therapy, has been strongly associated with poor prognosis in patients with surgically resectable CRC^10–12^. In this large prospective, observational study with 1,039 patients with clinical stage II to IV or recurrent CRC who underwent radical surgery (median follow-up of 16.74 months), we confirmed that patients with ctDNA positivity 4 weeks after surgery have a significantly higher risk of recurrence than those with ctDNA negativity 4 weeks after surgery (Fig. 2a). In this study, the choice of the 4-week postsurgical time point for MRD analysis was based on previous studies in which ctDNA levels immediately after surgery or within 4 weeks after surgery were observed to be masked owing to surgery-induced increases in cell-free DNA levels^13^. Moreover, most patients begin their ACT treatment between 6 weeks and 8 weeks after surgery. Furthermore, except for *BRAF* and *RAS* status, ctDNA was observed to be the most significant risk factor for recurrence in multivariate analysis (Fig. 2b). This is exemplified by the fact that ctDNA-positive patients 4 weeks after surgery with stage I and low-risk stage II CRC had markedly higher rates of recurrence than ctDNA-negative patients with resected oligometastatic CRC. We believe that these results can help to refine staging criteria in the future by incorporating postsurgical ctDNA status into traditional TNM staging criteria.

In this study, the tumor-informed, personalized ctDNA assay detected ctDNA before surgery in 91.3% (934 out of 1,023) of patients overall; specifically, in 94.5% (291 out of 308) of patients with pathological stage II disease, 97.2% (380 out of 391) of patients with stage III disease, and 84.2% (192 out of 228) of patients with stage IV oligometastatic or recurrent disease (Extended Data Fig. 3). It is important to note that the lower presurgical ctDNA detection rate for patients with clinical stage IV or recurrent CRC is probably due to prior chemotherapy and other factors, and suggests that NAC may obscure molecular disease in these patients (77.1% (27 out of 35) with prior chemotherapy versus 85.6% (166 out of 194) without chemotherapy).

Notably, 55.7% (4,664 out of 8,374) of genes (Extended Data Fig. 2) selected for the tumor-informed ctDNA assay were found to be unique to each patient. This highlights the importance of personalized ctDNA analysis based on patient-specific somatic tumor mutations.

Currently, ACT for patients with CRC after surgery is considered according to clinicopathological risk factors, including pathological TNM staging, lymphovascular invasion, clinical obstruction and perforation, particularly for patients with stage II disease^1,2^. However, it has been reported that standard ACT can decrease the absolute recurrence rate by only 10–15% in the overall population harboring clinicopathological risk factors, with side effects including oxaliplatin-induced peripheral neuropathy^14,15^. However, clinically, the decision to administer ACT is frequently weighed against factors that include patient preference, patient performance status and clinical risk of recurrence. Furthermore, in patients with oligometastatic stage IV colon adenocarcinoma, the benefit of ACT after surgical resection is inconsistent, especially when the patient may have received NAC. In addition, although improvement in progression-free survival has been demonstrated in several trials, this did not translate to an improvement in overall survival^16,17^.

Our findings improve on the current treatment paradigm. ctDNA positivity 4 weeks after surgery was observed to be a strong prognostic marker that identified a group of patients with a high risk of recurrence (18%; 187 out of 1,039) and inferior DFS (HR 10.0, *P* < 0.0001) (Fig. 2a). In addition, our study supports ctDNA positivity as a predictive marker of ACT benefit, which is exemplified by the overall benefit derived from ACT by ctDNA-positive patients versus by the observation group (Fig. 3a), an effect that was observed across all pathological stages after adjusting for confounding variables. Therefore, our data suggest that regardless of pathological stage, patients who are at a higher risk of relapse (based on ctDNA status) may benefit from ACT. Interestingly, analysis of data from patients with oligometastatic stage IV disease who may have previously received NAC showed that ACT was not associated with significant improvement in DFS. Instead, ACT benefit was more pronounced in patients who received upfront surgery (without NAC) (Extended Data Fig. 7a). Of note, the limited sample set of this analysis should be considered when interpreting these results. Overall, our findings demonstrate that ctDNA may improve upon patient selection for ACT. It also raises the question of whether patients who remain MRD positive after NAC should be referred to clinical trials given their poor prognosis.

Furthermore, our study showed that ACT can lead to increased rates of ctDNA clearance and can potentially modify outcomes of patients with postsurgical ctDNA positivity. A similar trend was observed in a recent study^18^. The authors performed a retrospective ctDNA analysis in a cohort of patients with muscle-invasive urothelial cancer who were part of a phase III IMvigor010 (NCT02450331) study. The patients were given adjuvant atezolizumab (ICI) as adjuvant treatment (*n* = 300) or were in the observation group (*n* = 281), and the median follow-up was 21.9 months (range 16–45 months). Patients with postsurgical ctDNA positivity benefited from atezolizumab (HR 0.59, *P* = 0.0059), whereas no such benefit was observed for ctDNA-negative patients (HR 1.14)^18^. Considering the Bradford Hill criteria for evaluating a potential causal relationship, the criterion ‘analogy’ suggests that strong evidence of a similar relationship should be taken into account, and here, the similar trend reported in urothelial cancer^18^ supports our results.

In this study, patients who were ctDNA positive after surgery who lacked ctDNA clearance had a markedly increased risk of recurrence. This opens the opportunity to use postsurgical ctDNA status or lack of clearance to enrich future studies with an aim to improve outcomes in this patient population. Currently, to improve prognosis in the ctDNA-positive population, we are conducting the ALTAIR trial, a phase III study to investigate the benefit of trifluridine/tipiracil versus placebo on DFS for CRC, in patients with ctDNA positivity with no evidence of clinical recurrence, even after standard-of-care treatment^9^. Meanwhile, we found that ctDNA negativity 4 weeks after surgery was correlated with favorable outcomes in patients with high-risk pathological stage II or stage III CRC regardless of ACT (Fig. 3b). Notably, an 18-month DFS of over 90% was observed in these patients (Fig. 3b).

Although limited by the length of follow-up, our study suggests that in patients who are ctDNA negative 4 weeks after surgery, observation alone may be sufficient for favorable patient outcomes. However, a longer follow-up would be needed to validate these findings and exclude a substantial benefit of ACT in these patients. Indeed, several prospective studies are investigating the omission of ACT for patients with ctDNA-negative status after surgery^19,20^. However, as the bias of patient characteristics is inevitable in observational studies, we are conducting a randomized, phase III VEGA trial to assess the noninferiority of observation alone compared with standard ACT in patients with high-risk stage II or low-risk pathological stage III CRC who are confirmed to be ctDNA negative 4 weeks after surgery.

Potential limitations of these results include the observational nature of the study and the bias in patient characteristics. To partly mitigate these potential biases, we performed a multivariate analysis, demonstrating the clear benefit of ACT for reduction of recurrence risk in ctDNA-positive patients 4 weeks after surgery. Another limitation of the study is the nonfeasibility of conducting a randomized trial of ACT versus the observation arm in postsurgical ctDNA-positive patients in Japan. However, our results are supportive of the benefit of ACT and may need further investigation in a prospective randomized trial with adequate follow-up to evaluate the noninferiority of the observation arm versus ACT in ctDNA-negative patients. Our data are supported by the results of the recently published prospective randomized DYNAMIC trial^22^. Of 455 patients with stage II colon cancer, 294 underwent ctDNA-guided adjuvant therapy, and 147 underwent standard management (median follow-up of 37 months). ctDNA-guided management reduced the proportion of patients receiving adjuvant therapy (15% in the ctDNA-guided arm versus 28% in the standard management arm), without compromising 2-year recurrence-free survival (93.5% in the ctDNA-guided arm versus 92.4% in the standard management arm), implying that ctDNA-guided ACT is not inferior to standard management. Interestingly, patients who were ctDNA negative were not treated, and the 3-year recurrence-free survival for ctDNA-negative patients was 92.5% compared with 86.4% for ctDNA-positive patients^22^.

In conclusion, we highlight the prognostic role of a personalized and tumor-informed ctDNA assay in patients with surgically resectable CRC. In this large observational study, we demonstrate that postsurgical ctDNA status is a most significant prognostic biomarker than the currently used high-risk clinicopathological features and can potentially be predictive of ACT benefit. Ongoing prospective randomized trials will further investigate the optimal ctDNA-guided treatment strategy for surgically resectable CRC.

## Methods

### Study design and participants

Here we present the interim analysis from the GALAXY cohort, the observational arm of the ongoing prospective and multicenter CIRCULATE-Japan study. The GALAXY study is a prospective large-scale nationwide registry designed to monitor ctDNA status for patients with clinical stage II to IV CRC who can undergo complete surgical resection. It serves to screen patients for ctDNA-guided MRD status, leading to their assignment to one of the two randomized ctDNA-guided interventional phase III trials, known as ALTAIR (treatment escalation) and VEGA (treatment de-escalation). The ALTAIR study evaluates the efficacy and safety of preemptive treatment with trifluridine/tipiracil compared with standard-of-care. Patients who test ctDNA positive after undergoing curative resection in GALAXY will be recruited into ALTAIR and randomly assigned to treatment or control. VEGA tests noninferiority of observation versus adjuvant capecitabine plus oxaliplatin. These trials incorporate a crossover component in which VEGA participants who become ctDNA positive can enter the ALTAIR trial. The study protocol has previously been published^7^, and the study design is presented in Fig. 1a. Written informed consent was obtained from all patients before participation in the study. The clinical protocol was approved by the Institutional Review Board of the National Cancer Center Japan and authorized by the head of each participating institution. The names of all participating institutions are provided in Supplementary Table 4. The study has been registered in the Japan Registry of Clinical Trials (UMIN000039205) and was conducted in accordance with the Declaration of Helsinki.

Between 5 June 2020 and 30 April 2021, a total of 1,563 patients with clinical stage II or III colon cancer or surgically resectable clinical stage IV or recurrent CRC were prospectively enrolled in the GALAXY study at 92 institutions. This study presents an interim analysis of the results of GALAXY with a median follow-up of 16.74 months (range 0.49–24.83 months) as of 8 June 2022. The data cutoff for this interim analysis was planned for the first 1,500 patients enrolled to be statistically relevant and clinically impactful. This is an ongoing study with an expected enrollment of over 5,000 patients. Key eligibility criteria included the following: histologically confirmed colorectal adenocarcinoma; primary location of the tumor as the colon or rectum (excluding appendix and anal canal cancer); curative resection planned for clinical stage II or III in the Union for International Cancer Control (eighth edition), or R0 resection planned for relapsed or stage IV CRC; age ≥ 20 years; and Eastern Cooperative Oncology Group performance status 0–1. Patients with other malignancies diagnosed within 5 years were excluded.

Of the 1,563 patients, 524 were excluded from the ctDNA analysis based on the following criteria: enrollment in one of the interventional CIRCULATE cohorts (*n* = 289; Supplementary Table 5); absence of ctDNA results at the 4-week postsurgical time point (*n* = 103); unknown pathological stage (*n* = 107); incomplete resection or noncurative surgery (*n* = 19); withdrawal of informed consent (*n* = 2); and pathological stage 0 (*n* = 4) (Extended Data Fig. 1b). A total of 7,285 plasma samples from 1,039 patients were included in this analysis. Blood samples were collected before surgery and 4 weeks, 12 weeks, 24 weeks, 36 weeks, 48 weeks and 72 weeks after surgery with concurrent computed tomography imaging performed every 6 months after surgery. In addition, CEA measurements were performed as per the study protocol. *RAS* and *BRAF*^V600E^ mutational status and MSI were measured using the MEBGEN RASKET-B KIT (Medical & Biological Laboratories) and polymerase chain reaction-based MSI test (SRL) at the central laboratory. Data collection was performed by input into an electronic data capture system (TrialMaster v5.0 (update 6), Anju Life Sciences Software).

Our study is a clinical study with human participants with a median age of 69 years (range 25–93 years), who self-reported their biological sex on the requisition form upon enrollment. Information on the breakdown of biological sex in our cohort is detailed in Table 1, in which 52.9% (550 out of 1,039) of the cohort were male and the remainder were female. Finally, biological sex was one variable analyzed in our multivariate analysis. Raw data for patient characteristics are provided in Supplementary Table 6.

### Personalized ctDNA assay for MRD detection

Formalin-fixed, paraffin-embedded (FFPE) tumor tissue samples from surgical resection or biopsy were used for WES to identify up to 16 patient-specific clonal, somatic single-nucleotide variants (SNVs), as previously described^10^. These SNVs were used to design personalized multiplex polymerase chain reaction-based next-generation sequencing assays (Signatera, Natera) for each study participant. Cell-free DNA was extracted from patient plasma (median 9.9 ml, range 1.6–12.2 ml), at a given time point and was used to detect ctDNA. Plasma samples with at least 2 out of 16 tumor-specific variants detected above a predefined threshold were defined as ctDNA positive. The predefined threshold is based on Natera’s proprietary variant calling method wherein detecting at least 2 out of 16 variants ensures the optimal analytical performance of the assay with > 95% sensitivity at 0.01% mean variant allele frequency and with 99.7% specificity^21^. ctDNA concentration was reported in mean tumor molecules per ml of plasma. ctDNA results for eligible patients before surgery and 4 weeks and 12 weeks after surgery are provided in Supplementary Table 7.

### Statistical analysis

The primary endpoint was DFS, which is defined as the time between the date of surgery and date of diagnosis with relapse or death due to any cause. Relapse was determined based on diagnostic imaging or any other diagnostic procedure if imaging was not confirmative (that is, colonoscopy to diagnose local recurrence). The chi-squared test was used to compare categorical variables. Survival analyses were carried out using R software v3.6.1 using packages *survival* and *survminer*. The Kaplan–Meier method was used to estimate the survival distribution. Differences between the groups were tested using the log-rank test. A multivariable Cox proportional hazards model was used to assess prognostic factors associated with DFS (coxph and cox.zph). Clinically relevant cutoffs were applied for demographic variables, wherever appropriate. To account for immortal time bias, a landmark analysis was performed 8 weeks after surgery for cohorts that evaluated the effect of ACT, whereby DFS was measured starting from day 60. Landmark analysis was also used to evaluate the association of changes in postsurgical ctDNA status from week 4 to week 12 with DFS. To account for the immortal time bias, patients who were alive until at least 12 weeks after surgery were included in the dynamics analysis. The secondary endpoint was ctDNA clearance analysis, which was performed using SAS software v9.4, and Gray’s test was used to compare cumulative incidence function differences between the ACT and observation groups^23^. Analysis of ctDNA concentration across stages and at different time points was performed using ggplot2 package v3.3.6 in R v4.2.1. *P* values < 0.05 was considered statistically significant.

### Reporting summary

Further information on research design is available in the Nature Portfolio Reporting Summary linked to this article.

## Online content

Any methods, additional references, Nature Portfolio reporting summaries, source data, extended data, supplementary information, acknowledgements, peer review information; details of author contributions and competing interests; and statements of data and code availability are available at 10.1038/s41591-022-02115-4.

## Supplementary information

Supplementary Information Supplementary Tables 1–3

Reporting Summary

Supplementary Tables Supplementary Table 4 Names of participating institutes. Supplementary Table 5 Baseline characteristics of 289 patients who were excluded owing to enrollment in phase III clinical trials. Supplementary Table 6 Raw data representing patient characteristics. Supplementary Table 7 Raw data of ctDNA results before surgery, and 4 weeks and 12 weeks after surgery.

## Data Availability

The authors declare that all relevant data used to conduct the analyses are available within the article. To protect the privacy and confidentiality of patients in this study, clinical data are not made publicly available in a repository or the supplementary material of the article but can be requested at any time from the corresponding author. Any requests will be reviewed within a time frame of 2 to 3 weeks by the CIRCULATE-Japan study steering committee to verify whether the request is subject to any intellectual property or confidentiality obligations. All data shared will be de-identified.

## Code availability

The fully documented code for the R statistical computing environment for analyses related to this manuscript are deposited at the github repository and can be accessed at https://github.com/ssharma-natera/Nature_Medicine_Eiji.
