# Supplementary material for: Molecular residual disease and efficacy of adjuvant chemotherapy in patients with colorectal cancer
Source: Nat Med. 2023 Jan 16;29(1):127–34. doi: 10.1038/s41591-022-02115-4 (PMC9873552; doi:10.1038/s41591-022-02115-4)
Supplement: Supplementary file 1 — Supplementary Tables 1–3 [file 41591_2022_2115_MOESM1_ESM.pdf]

nature medicine

Article

https://doi.org/10.1038/s41591-022-02115-4

Molecular residual disease and efficacy of adjuvant chemotherapy in patients with colorectal cancer

In the format provided by the authors and unedited

Molecular Residual Disease and Efficacy of Adjuvant Chemotherapy in Patients with Clinical Stage II-IV Colorectal Cancer

Daisuke Kotani[†,1], Eiji Oki[†,2], Yoshiaki Nakamura[†1,3], Hiroki Yukami[1,4], Saori Mishima[1], Hideaki Bando[1,3], Hiromichi Shirasu[5], Kentaro Yamazaki[5], Jun Watanabe[6], Masahito Kotaka[7], Keiji Hirata[8], Naoya Akazawa[9], Kozo Kataoka,[10], Shruti Sharma[11], Vasily N. Aushev[11], Alexey Aleshin[11], Toshihiro Misumi[12], Hiroya Taniguchi[13], Ichiro Takemasa[14], Takeshi Kato[15], Masaki Mori[16], Takayuki Yoshino[1]

Affiliations

1. Department of Gastroenterology and Gastrointestinal Oncology, National Cancer Center Hospital East, Kashiwa, Japan

2. Department of Surgery and Science, Graduate School of Medical Science, Kyushu University, Fukuoka, Japan

3. Translational Research Support Section, National Cancer Center Hospital East, Kashiwa, Japan

4. The Second Department of Internal Medicine Center, Osaka Medical and Pharmaceutical University, Takatsuki, Japan.

5. Division of Gastrointestinal Oncology, Shizuoka Cancer Center, Sunto-gun, Japan

6. Department of Surgery, Gastroenterological Center, Yokohama City University Medical Center, Yokohama, Japan

7. Gastrointestinal Cancer Center, Sano Hospital, Kobe, Japan

8. Department of Surgery 1, School of Medicine, University of Occupational and Environmental Health, Kitakyushu, Japan

9. Department of Gastroenterological Surgery, Sendai City Medical Center Sendai Open Hospital, Sendai, Japan

10. Division of Lower GI Surgery, Department of Surgery, Hyogo College of Medicine, Hyogo, Japan.

11. Natera, Inc., Austin, TX, U.S.A.

12. Department of Data Science, National Cancer Center Hospital East, Kashiwa, Japan

13. Department of Clinical Oncology, Aichi Cancer Center, Nagoya, Japan

14. Department of Surgery, Surgical Oncology and Science, Sapporo Medical University, Sapporo, Japan

15. Department of Surgery, National Hospital Organization Osaka National Hospital, Osaka, Japan

16. Tokai University School of Medicine, Isehara, Japan

† These authors contributed equally to this work.

Corresponding author

Eiji Oki

Department of Surgery and Science, Graduate School of Medical Science, Kyushu University, Fukuoka, Japan

Email: oki.eiji.857@m.kyushu-u.ac.jp

Supplementary Tables

Supplementary Table 1. Baseline characteristics of high-risk stage II and III patients with post-surgical ctDNA negative result (4-week) further stratified by ACT status

High-risk stage II-III patients with postsurgical ctDNA-negative status		Adjuvant chemotherapy N= 219 (%)	No-Adjuvant Chemotherapy (Observation) N=312 (%)	P-value**
Age	Median (range)	66 (25-89)	71 (44-93)	-
	< 70 years old	152 (69.4)	142 (45.5)	0.0001
	>70 years old	67 (30.6)	170 (54.5)	
Sex	Male	109 (49.8)	156 (50)	0.96
	Female	110 (50.2)	156 (50)	
ECOG PS	0	200 (91.3)	254 (81.4)	0.001
	1	19 (8.7)	58 (18.6)	
Primary location	Right-sided colon	90 (41.1)	145 (46.5)	0.35
	Left-sided colon	121 (55.3)	160 (51.3)	
	Rectum	8 (3.6)	7 (2.2)	
Pathological T stage	T1-2	9 (4.1)	8 (2.6)	0.31
	T3-4	210 (95.9)	304 (97.4)	
Pathological N stage	N0	39 (17.8)	194 (62.2)	0.0001
	N1-2	180 (82.2)	118 (37.8)	
Pathological stage	High risk Stage II	38 (17.3)	187 (60)	0.0001
	Stage III	181 (82.7)	125 (40)	

RAS/BRAF status	RAS/BRAF wild-type	108 (49.3)	149 (48.1)	0.95
	RAS mutant	87 (39.7)	125 (40.3)	
	BRAF mutant	24 (11)	36 (11.6)	
MSI status	MSI-High	21 (9.6)	52 (16.7)	0.01
	MSS	198 (90.4)	260 (83.3)	

**The p-values are calculated using the two-sided chi-square test that compares the distribution of the factors between the two columns (ACT vs Observation) with no correction for multiplicity.

Supplementary Table 2. Baseline characteristics of high-risk stage II and III patients with post-surgical ctDNA positive result (4-week) further stratified by ACT status

High-risk stage II-III patients with postsurgical ctDNA-positive status		Adjuvant chemotherapy N=72 (%)	No-Adjuvant chemotherapy (Observation) N=41 (%)	P-value**
Age	Median (range)	67.5 (39-85)	73 (42-88)	-
	< 70 years old	44 (61.1)	15 (36.6)	0.01
	>70 years old	28 (38.9)	26 (63.4)	
Sex	Male	40 (55.6)	30 (73.2)	0.06
	Female	32 (44.4)	11 (26.8)	
ECOG PS	0	65 (90.3)	30 (73.2)	0.01
	1	7 (9.7)	11 (76.8)	
Primary location	Right-sided colon	29 (40.3)	14 (34.1)	0.76
	Left-sided colon	42 (58.3)	26 (63.4)	

	Rectum	1 (1.4)	1 (2.5)	
Pathological T stage	T1-2	5 (7)	0 (0)	0.08
	T3-4	67 (93)	41 (100)	
Pathological N stage	N0	10 (13.9)	12 (29.3)	0.05
	N1-2	62 (86.1)	29 (70.7)	
Pathological stage	Stage II	9 (12.5)	13 (31.7)	0.01
	Stage III	63 (87.5)	28 (68.3)	
RAS/BRAF status	RAS/BRAF wild-type	35 (49.3)	21 (51.2)	0.23
	RAS mutant	35 (49.3)	17 (41.5)	
	BRAF mutant	1 (1.4)	3 (7.3)	
MSI status	MSI-High	2 (2.8)	2 (4.9)	0.57
	MSS	69 (97.2)	39 (95.1)	

**The p-values are calculated using the two-sided chi-square test that compares the distribution of the factors between the two columns (ACT vs Observation) with no correction for multiplicity.

Supplementary Table 3. Clearance Cohort: Baseline characteristics of patients with post-surgical ctDNA positive result (4-week) who either received or did not receive adjuvant chemotherapy

Patient Characteristics		Adjuvant chemotherapy N=92 (50.5%)	No adjuvant chemotherapy (observation) N=90 (49.5%)	P-value*
Age	Median (range)	67 (39-85)	68 (42-88)	-
	≤ 70	60 (65.2)	51 (56.7)	0.23
	>70 years old	32 (34.8)	39 (43.3)	
Sex	Male	49 (53.2)	65 (72.2)	0.008
	Female	43 (46.8)	25 (27.8)	
ECOG PS	0	85 (92.4)	75 (83.3)	0.06
	1	7 (7.6)	15 (16.7)	
Primary location	Right-sided colon	33 (35.8)	19 (21.6)	0.001
	Left-sided colon	56 (61.0)	53 (60.2)	
	Rectum	3 (3.2)	16 (18.2)	
Pathological T stage	T1-2	5 (6.0)	1 (2.0)	0.26
	T3-4	78 (94.0)	51 (98.0)	
Pathological N stage	N0	14 (16.8)	16 (30.8)	0.058
	N1-2	69 (83.2)	36 (69.2)	
Pathological stage	Stage I	0 (0)	1 (1.1)	

	Stage II	9 (9.8)	14 (15.6)	<0.001
	Stage III	63 (68.5)	27 (30.0)	
	Stage IV or recurrence	20 (21.7)	48 (53.3)	
RAS/BRAF status	RAS/BRAF wild-type	46 (50.6)	46 (51.7)	0.65
	RAS mutant	43 (47.2)	39 (43.8)	
	BRAF mutant	2 (2.2)	4 (4.5)	
MSI status	MSI-High	2 (2.2)	3 (3.4)	0.63
	MSS	89 (97.8)	86 (96.6)	

**The p-values are calculated using the two-sided chi-square test that compares the distribution of the factors between the two columns (ACT vs Observation) with no correction for multiplicity.
