# Supplementary material for: Molecular residual disease and efficacy of adjuvant chemotherapy in patients with colorectal cancer
Source: Nat Med. 2023 Jan 16;29(1):127–34. doi: 10.1038/s41591-022-02115-4 (PMC9873552; doi:10.1038/s41591-022-02115-4)
Supplement: Supplementary file 2 — Reporting Summary [file 41591_2022_2115_MOESM2_ESM.pdf]

Corresponding author(s): Eiji Oki

Last updated by author(s): Oct 7, 2022

Reporting Summary

Nature Portfolio wishes to improve the reproducibility of the work that we publish. This form provides structure for consistency and transparency in reporting. For further information on Nature Portfolio policies, see our Editorial Policies and the Editorial Policy Checklist.

Statistics

For all statistical analyses, confirm that the following items are present in the figure legend, table legend, main text, or Methods section.

n/a	Confirmed	
☐	☒	The exact sample size (*n*) for each experimental group/condition, given as a discrete number and unit of measurement
☒	☐	A statement on whether measurements were taken from distinct samples or whether the same sample was measured repeatedly
☐	☒	The statistical test(s) used AND whether they are one- or two-sided<br>*Only common tests should be described solely by name; describe more complex techniques in the Methods section.*
☐	☒	A description of all covariates tested
☐	☒	A description of any assumptions or corrections, such as tests of normality and adjustment for multiple comparisons
☐	☒	A full description of the statistical parameters including central tendency (e.g. means) or other basic estimates (e.g. regression coefficient) AND variation (e.g. standard deviation) or associated estimates of uncertainty (e.g. confidence intervals)
☐	☒	For null hypothesis testing, the test statistic (e.g. *F*, *t*, *r*) with confidence intervals, effect sizes, degrees of freedom and *P* value noted<br>*Give P values as exact values whenever suitable.*
☒	☐	For Bayesian analysis, information on the choice of priors and Markov chain Monte Carlo settings
☒	☐	For hierarchical and complex designs, identification of the appropriate level for tests and full reporting of outcomes
☒	☐	Estimates of effect sizes (e.g. Cohen's *d*, Pearson's *r*), indicating how they were calculated

Our web collection on statistics for biologists contains articles on many of the points above.

Software and code

Policy information about availability of computer code

Data collection	Data collection was done by input into an EDC systems; the EDC was TrialMaster version 5.0 (update 6) from Anju Life Sciences software.
Data analysis	Chi-square test was used to compare categorical variables. Survival analyses were carried out using R software version 3.6.1 using packages survival and survminer. The Kaplan–Meier method was used to estimate the survival distribution. Differences between the groups were tested by the log-rank test. A multivariable cox proportional hazards model was used to assess prognostic factors associated with DFS (coxph and cox.zph). Clinically relevant cutoffs were applied for demographic variables wherever appropriate. To account for immortal time bias, a landmark analysis was performed at 8 weeks for cohorts that evaluated the effect of ACT, whereby DFS was measured starting from day 60. Landmark analysis was also used to evaluate the association of ctDNA dynamics from 4-weeks to 12-weeks with DFS. To account for the immortal time bias, patients who were alive until at least 12 weeks were included in the dynamics analysis. ctDNA clearance analysis was performed using SAS software version 9.4 and Gray's test was used to compare cumulative incidence function differences between the ACT and observation groups. Analysis of ctDNA concentration across stages and at different time points was performed using ggplot2 package v3.3.6 in R v4.2.1. All P values <0.05 were considered statistically significant.

For manuscripts utilizing custom algorithms or software that are central to the research but not yet described in published literature, software must be made available to editors and reviewers. We strongly encourage code deposition in a community repository (e.g. GitHub). See the Nature Portfolio guidelines for submitting code & software for further information.

Data

Policy information about availability of data

All manuscripts must include a data availability statement. This statement should provide the following information, where applicable:

- Accession codes, unique identifiers, or web links for publicly available datasets
- A description of any restrictions on data availability
- For clinical datasets or third party data, please ensure that the statement adheres to our policy

Data availability: The authors declare that all relevant data used in the conduct of the analyses are available within the article. To protect the privacy and confidentiality of patients in this study, clinical data are not made publicly available in a repository or the supplementary material of the article but will be available at any time upon reasonable request to the Corresponding author. Those requests will be reviewed by a study steering committee to verify whether the request is subject to any intellectual property or confidentiality obligations. All data shared will be de-identified.
Code availability: The fully documented code for the R statistical computing environment for analyses related to this manuscript are deposited at the github repository and can be accessed at: https://github.com/ssharma-natera/Nature_Medicine_Eiji

Human research participants

Policy information about studies involving human research participants and Sex and Gender in Research.

Reporting on sex and gender	Our study is a clinical study with human participants who self-reported their biological sex on the requisition form upon enrollment. Information on the breakdown of biological sex in our cohort is detailed in Table 1 where 52.9% (550/1,039) of the cohort were male and the remainder were female. Finally, biological sex was one variable analyzed in our multivariate analysis.
Population characteristics	Population characteristics are provided in Table 1.
Recruitment	All registration was done on a voluntary basis by researchers at each participating site. The possibility of bias in the selection by researchers cannot be ruled out. However, since enrollment was prospective and each site enrolled most of the cases that fit the criteria, we believe that bias was suppressed as much as possible.
Ethics oversight	Written Informed consent was obtained from all patients before participating in the study. The clinical protocol was approved by the Institutional Review Boards at the National Cancer Center Japan and authorized by the head of each participating institution.

Note that full information on the approval of the study protocol must also be provided in the manuscript.

Field-specific reporting

Please select the one below that is the best fit for your research. If you are not sure, read the appropriate sections before making your selection.

☒ Life sciences ☐ Behavioural & social sciences ☐ Ecological, evolutionary & environmental sciences

For a reference copy of the document with all sections, see nature.com/documents/nr-reporting-summary-flat.pdf

Life sciences study design

All studies must disclose on these points even when the disclosure is negative.

Sample size	This is an interim analysis of the planned GALAXY study. The data cutoff for this interim analysis was planned for the first 1500 patients enrolled, to be statistically relevant and clinically impactful. No other sample size calculation was performed for the ctDNA analysis. The sample size was determined by the available by the number of patients who had evaluable tissue available for whole exome sequencing and blood samples at 4 and 12 weeks.
Data exclusions	Patients with other malignancies diagnosed within 5 years were excluded. Of the 1,563 patients, 524 patients were excluded from the ctDNA analysis based on the following criteria: a) enrollment in one of the interventional CIRCULATE cohorts (n=289, Supplementary Table 1), b) absence of ctDNA results at the 4-week postoperative time point (n=103), c) unknown pathological stage (n=107), d) incomplete resection or non-curative surgery (n=19), e) withdrawal of informed consent (n=2), and f) pathological stage 0 (n=4) (Extended Data Fig. 1B). For dynamics analysis, patients who did not have ctDNA results at 12-weeks after surgery were excluded (n=202) and who recurred within 12 weeks (n=45) were excluded. For clearance analysis, patients with no subsequent ctDNA results available (n=5) were excluded. For ctDNA-negative cohort that analyzed patients with high-risk stage II and stage III, patients who had pathological-stage IV or recurrence (n=160), pathological stage I (n=95) and pathological low-risk stage II (n=66) were excluded. Complete detail on exclusions is provided in Figure 1B.
Replication	Due to sample size and biospecimen availability, ctDNA measurements were performed once on each sample and were not repeated. Note that the ctDNA assay run on the patient samples has been analytically validated previously.

Randomization	This is an observational study and is not a randomized trial.
Blinding	The ctDNA measurements were conducted by Natera in a manner that kept them blinded to clinical data including PFS, OS, and objective response. All treatment and clinical management decisions were made blinded to ctDNA results. All clinical outcome data were collected by individuals blinded to the ctDNA results.

Reporting for specific materials, systems and methods

We require information from authors about some types of materials, experimental systems and methods used in many studies. Here, indicate whether each material, system or method listed is relevant to your study. If you are not sure if a list item applies to your research, read the appropriate section before selecting a response.

Materials & experimental systems

n/a	Involved in the study
☒	☐ Antibodies
☒	☐ Eukaryotic cell lines
☒	☐ Palaeontology and archaeology
☒	☐ Animals and other organisms
☐	☒ Clinical data
☒	☐ Dual use research of concern

Methods

n/a	Involved in the study
☒	☐ ChIP-seq
☒	☐ Flow cytometry
☒	☐ MRI-based neuroimaging

Clinical data

Policy information about clinical studies
All manuscripts should comply with the ICMJE guidelines for publication of clinical research and a completed CONSORT checklist must be included with all submissions.

Clinical trial registration	The study has been registered in the University hospital Medical Information Network (UMIN000039205).
Study protocol	The study protocol is submitted along with the manuscript.
Data collection	All data collection was done by input into an EDC systems; the EDC was TrialMaster version 5.0 (update 6) from Anju Life Sciences software.
Outcomes	A statistical analysis plan was designed prior to data analysis. The primary endpoint was disease-free survival (DFS), defined as the time between the date of the surgery and the date of diagnosis with relapse or death due to any cause. Relapse was determined based on diagnostic imaging or any other diagnostic procedure if imaging was not confirmative (i.e. colonoscopy to diagnose local recurrence). Chi-square test was used to compare categorical variables. Survival analyses were carried out using R software version 3.6.1 using packages survival and survminer. The Kaplan–Meier method was used to estimate the survival distribution. Differences between the groups were tested by the log-rank test. A multivariable cox proportional hazards model was used to assess prognostic factors associated with DFS (coxph and cox.zph). Clinically relevant cutoffs were applied for demographic variables wherever appropriate. To account for immortal time bias, a landmark analysis was performed at 8 weeks for cohorts that evaluated the effect of ACT, whereby DFS was measured starting from day 60. Landmark analysis was also used to evaluate the association of ctDNA dynamics from 4-weeks to 12-weeks with DFS. To account for the immortal time bias, patients who were alive until at least 12 weeks were included in the dynamics analysis.<br>The secondary endpoint was ctDNA clearance analysis, which was performed using SAS software version 9.4 and Gray's test was used to compare cumulative incidence function differences between the ACT and observation groups. Analysis of ctDNA concentration across stages and at different time points was performed using ggplot2 package v3.3.6 in R v4.2.1. All P values <0.05 were considered statistically significant.
